# Supplementary material for: English major students’ satisfaction with ELSA Speak in English pronunciation courses
Source: PLoS One. 2025 Jan 9;20(1):e0317378. doi: 10.1371/journal.pone.0317378 (PMC11717304; doi:10.1371/journal.pone.0317378)
Supplement: S1 File — (DOCX) [file pone.0317378.s001.docx]

**QUESTIONNAIRE SURVEY**

**APPENDIX 1**

We are conducting a study on your expectations before using the ELSA SPEAK application to develop English pronunciation skills, and your level of satisfaction after using this application. Your answers are a valuable source of data that helps us have the most objective assessments and appropriate directions for using the ELSA SPEAK application to improve students' pronunciation in the future. Thank you very much

**PERSONAL INFORMATION SECTION**

1. Gender: Male 🞏 Female 🞏 School-year student: 1 🞏 2 🞏 3 🞏

2. Have you ever practiced on ELSA SPEAK before? Yes 🞏 No 🞏

Please mark (**X**) at one of the following levels: 1 (**Strongly disagree**), 2 (**Disagree**), 3 (**Neutral**), 4 (**Agree**), 5 (**Strongly agree**).

| **No.** | **Code** | **Items** | **1** | **2** | **3** | **4** | **5** |
| --- | --- | --- | --- | --- | --- | --- | --- |
| **1** | PU1 | I believe that ELSA Speak can help me pronounce single vowels accurately. |  |  |  |  |  |
| **2** | PU2 | I believe that ELSA Speak can help me connect sounds between words in sentences better. |  |  |  |  |  |
| **3** | PU3 | I believe that ELSA Speak can help me pronounce diphthongs accurately. |  |  |  |  |  |
| **4** | PU4 | I believe that ELSA Speak can help me pronounce voiceless consonants accurately. |  |  |  |  |  |
| **5** | PU5 | I believe that ELSA Speak can help me pronounce voiced consonants accurately. |  |  |  |  |  |
| **6** | PU6 | I believe that ELSA Speak can help me stress words accurately. |  |  |  |  |  |
| **7** | PU7 | I believe that ELSA Speak can help me have better intonation when speaking English. |  |  |  |  |  |
| **8** | PEU1 | I believe that operations on ELSA Speak are easy to perform. |  |  |  |  |  |
| **9** | PEU2 | I believe that practicing on ELSA Speak does not require too many operations. |  |  |  |  |  |
| **10** | PEU3 | I believe that necessary features are fully supported on ELSA Speak so that I can do the exercises easily. |  |  |  |  |  |
| **11** | C1 | Practicing on ELSA Speak helped me pronounce single vowels more accurately. |  |  |  |  |  |
| **12** | C2 | Practicing on ELSA Speak helped me to connect sounds between words in sentences better. |  |  |  |  |  |
| **13** | C3 | Practicing on ELSA Speak helped me pronounce diphthongs more accurately. |  |  |  |  |  |
| **14** | C4 | Practicing on ELSA Speak helped me pronounce voiceless consonants more accurately. |  |  |  |  |  |
| **15** | C5 | Practicing on ELSA Speak helped me pronounce voiced consonants more accurately. |  |  |  |  |  |
| **16** | C6 | Practicing on ELSA Speak helped me stress words more accurately. |  |  |  |  |  |
| **17** | C7 | Practicing on ELSA Speak helped me have better intonation when speaking English. |  |  |  |  |  |
| **18** | C8 | The operations on ELSA Speak were easy to perform for me. |  |  |  |  |  |
| **19** | C9 | I didn’t have to do too many operations to practice on ELSA Speak. |  |  |  |  |  |
| **20** | C10 | ELSA Speak fully supported necessary features, so I could practice the exercises easily. |  |  |  |  |  |
| **21** | S1 | I will continue to use ELSA Speak in the future. |  |  |  |  |  |
| **22** | S1 | I will regularly practice pronunciation on ELSA Speak. |  |  |  |  |  |
| **23** | S3 | I will recommend ELSA Speak to others to use. |  |  |  |  |  |

**APPENDIX 2**

**QUESTIONNAIRE SURVEY (ORIGINAL VERSION)**

We are conducting a study on your expectations before using the ELSA SPEAK application to develop English pronunciation skills, and your level of satisfaction after using this application. Your answers are a valuable source of data that helps us have the most objective assessments and appropriate directions for using the ELSA SPEAK application to improve students' pronunciation in the future. Thank you very much

**PERSONAL INFORMATION SECTION**

1. Gender: Male 🞏 Female 🞏 School-year student: 1 🞏 2 🞏 3 🞏

2. Have you ever practiced on ELSA SPEAK before? Yes 🞏 No 🞏

Please mark (**X**) at one of the following levels: 1 (**Strongly disagree**), 2 (**Disagree**), 3 (**Neutral**), 4 (**Agree**), 5 (**Strongly agree**).

| **No.** | **Clusters** | **Items** | **1** | **2** | **3** | **4** | **5** |
| --- | --- | --- | --- | --- | --- | --- | --- |
| **1** | Perceived usefulness | I believe that ELSA Speak can help me pronounce single vowels accurately. |  |  |  |  |  |
| **2** |  | I believe that ELSA Speak can help me connect sounds between words in sentences better. |  |  |  |  |  |
| **3** |  | I believe that ELSA Speak can help me pronounce diphthongs accurately. |  |  |  |  |  |
| **4** |  | I believe that ELSA Speak can help me pronounce voiceless consonants accurately. |  |  |  |  |  |
| **5** |  | I believe that ELSA Speak can help me pronounce voiced consonants accurately. |  |  |  |  |  |
| **6** |  | I believe that ELSA Speak can help me stress words accurately. |  |  |  |  |  |
| **7** |  | I believe that ELSA Speak can help me have better intonation when speaking English. |  |  |  |  |  |
| **8** | Perceived ease of use | I believe the practice content of the application will fit into my curriculum. |  |  |  |  |  |
| **9** |  | I believe that the instructions on the application is presented clearly. |  |  |  |  |  |
| **10** |  | I believe that the instructions on the application is easy to understand for me. |  |  |  |  |  |
| **11** |  | I believe that I will get accurate feedback from the application for my practice. |  |  |  |  |  |
| **12** |  | I believe that operations on ELSA Speak are easy to perform. |  |  |  |  |  |
| **13** |  | I believe that practicing on ELSA Speak does not require too many operations. |  |  |  |  |  |
| **14** |  | I believe that necessary features are fully supported on ELSA Speak so that I can do the exercises easily. |  |  |  |  |  |
| **15** | Confirmation | Practicing on ELSA Speak helped me pronounce single vowels more accurately. |  |  |  |  |  |
| **16** |  | Practicing on ELSA Speak helped me to connect sounds between words in sentences better. |  |  |  |  |  |
| **17** |  | Practicing on ELSA Speak helped me pronounce diphthongs more accurately. |  |  |  |  |  |
| **18** |  | Practicing on ELSA Speak helped me pronounce voiceless consonants more accurately. |  |  |  |  |  |
| **19** |  | Practicing on ELSA Speak helped me pronounce voiced consonants more accurately. |  |  |  |  |  |
| **20** |  | Practicing on ELSA Speak helped me stress words more accurately. |  |  |  |  |  |
| **21** |  | Practicing on ELSA Speak helped me have better intonation when speaking English. |  |  |  |  |  |
| **22** |  | The practice content of the application fitted into my curriculum. |  |  |  |  |  |
| **23** |  | The instructions on the application were presented clearly. |  |  |  |  |  |
| **24** |  | The instructions on the application were easy to understand for me. |  |  |  |  |  |
| **25** |  | I got accurate feedback from the application for my practice. |  |  |  |  |  |
| **26** |  | The operations on ELSA Speak were easy to perform for me. |  |  |  |  |  |
| **27** |  | I didn’t have to do too many operations to practice on ELSA Speak. |  |  |  |  |  |
| **28** |  | ELSA Speak fully supported necessary features, so I could practice the exercises easily. |  |  |  |  |  |
| **29** | Satisfaction | I will continue to use ELSA Speak in the future. |  |  |  |  |  |
| **30** |  | I will regularly practice pronunciation on ELSA Speak. |  |  |  |  |  |
| **31** |  | I will recommend ELSA Speak to others to use. |  |  |  |  |  |
| **32** |  | I am satisfied with my learning experience on ELSA Speak. |  |  |  |  |  |
| **33** |  | I am satisfied with the practice content on ELSA Speak. |  |  |  |  |  |
| **34** |  | I am satisfied with the feedback from ELSA for my practice sections. |  |  |  |  |  |
