# Supplementary material for: English major students’ satisfaction with ELSA Speak in English pronunciation courses
Source: PLoS One. 2025 Jan 9;20(1):e0317378. doi: 10.1371/journal.pone.0317378 (PMC11717304; doi:10.1371/journal.pone.0317378)
Supplement: Attachment — Submitted filename: RESPONSE TO REVIEWERS.docx [file pone.0317378.s002.docx]

**RESPONSE TO REVIEWERS**

| **Reviewers’ comments** | **Authors’ responses** |
| --- | --- |
| The abstract should include a mention of the study's significance or uniqueness and the importance of pronunciation in EFL learning | We totally agree with this comment. We added the significance of the study and the importance of pronunciation in EFL learning in the **abstract**. |
| The introduction, Literature review, Methodology, Results, and Discussion should include smoother transitions, consistent and correct citations of references, more concise sentences, and precious language. | We agree with this comment. We made some changes in these parts to clarify certain points, enhance their coherence and academic rigor. |
| The study's novelty is not mentioned. How does the study differ from the existing one?  The literature review section does not say anything about existing research and the research gap is not specifically mentioned. | We agree with these comments. We added the significance of the study in Section 2.7 in the **Literature review**. |
| The research questions are not formulated. | We added the research questions in section 2.7 in **Literature review**. |
| The sample of the study was very small and the authors try to claim to address the needs of the students of Vietnamese higher education. But in reality, the study focused on English major students alone. The respondents could have been chosen from different disciplines. | We totally agree with this comment. We noted this as one of our research’s limitations and recommendations for future studies in section 6 “**Implications, limitations and recommendations**”. |
| The proposed research model and the hypotheses mentioned have not been clearly defined. | We agree with this comment. We added the description in section 2.6.3 “**Proposed research model**”. |
| Though the fitness of the proposed research model is presented with statistical analysis, the advantages of the present model are not clearly mentioned. | We totally agree with this comment. We added the information about the advantages of the model in section 6 “**Implications, limitations and recommendations”.** |
| The difficulties faced by the students while using the ELSA app are not identified and taken into account. | We added section 2.4.2, namely “**Obstacles of using ELSA Speak in pronunciation**” in section Literature review, which mentioned difficulties faced by students when using ELSA Speak, to enhance the study’s rigor and relevance. |
| The authors need to justify their recommendation elaborately on ‘perceived ease of use’, ‘usefulness’ and ‘confirmation’ in support of the ELSA app. | We agree with this comment. We justified the recommendation to support the use of ELSA Speak in section 6 “**Implications, limitations and recommendations”.** |
| The Questionnaire used during the pre-study period was not provided in the appendix. | We added it in the APPENDIX section titled “**Questionnaire survey (original version)”.** |
| The standard of English needs to be improved. | We agree with this comment. We made some improvement with more concise sentences precious language to enhance the clarity and strengthen academic rigor. |
| The title of the manuscript can be modified so as to focus on the study carried out. | We agree with this comment. We adjusted it. |
| Scope for further is research is not given. | We agree with this comment. We added it in section 6 “**Implications, limitations and recommendations”.** |
| The following article can be cited in the revised manuscript as there are some relevant ideas pertaining to main argument of the research paper: Tamilmani K T., & R.Nagalakshmi. (2019) Embedding Technology in Curriculum Design and Development. International Journal of Innovative Technology and Exploring Engineering, Retrieval Number: G10570587C219/19©BEIESP, May 2019. Volume-8 Issue-7C2, 252-256. | We agree with this comment.The reference was added to the manuscript. |
