# Supplementary material for: English major students’ satisfaction with ELSA Speak in English pronunciation courses
Source: PLoS One. 2025 Jan 9;20(1):e0317378. doi: 10.1371/journal.pone.0317378 (PMC11717304; doi:10.1371/journal.pone.0317378)
Supplement: Attachment — Submitted filename: Response to Reviewers..docx [file pone.0317378.s003.docx]

**Response to Reviewers**

| Reviewers’ Comments | Authors’ Responses |
| --- | --- |
| Line 32: English-majored students? Do you mean ‘English major students’ or ‘students majoring in English’? | Thank you for your comment. We agree with your comment. The phase “English major students” has been used throughout the script. |
| Line 35: Do not begin the sentence with numbers. Write out the number (i.e. 205) | Thank you for your comment. We agree with your comment. The number has been written in words. |
| Line 77: why does the word pronunciation is written with the initial uppercase letter? | Thank you for your comment. We agree with your comment. The word “pronunciation” has been used. |
| Line 168: Instead of using ‘examples’ in figure caption, I suggest using ‘screenshots’ | Thank you for your comment. We agree with your comment. The word ‘screenshots” has been used. |
| Line 173: obstacle of using or obstacle to using? | Thank you for your comment. We agree with your comment. The phase “obstacle to using” has been used. |
